# Refractory portal hypertension complications successfully managed by parallel transjugular intrahepatic portosystemic shunt (TIPS): a case report

**DOI:** 10.1186/s42155-022-00297-z

**Published:** 2022-04-18

**Authors:** Senali Weeratunga, Mithun Nambiar, Charles Handley, Cosmin Florescu, Stuart M. Lyon, Suong Le, Diederick W. De Boo

**Affiliations:** 1 Monash Imaging, Monash Medical Centre, Clayton, Victoria Australia; 2 Faculty of Medicine, Nursing and Health Science, Monash University, Clayton, Victoria Australia; 3 School of Clinical Sciences, Monash University, Clayton, Victoria Australia; 4 Department of Gastroenterology and HepatologyMonash Health, Clayton, Victoria Australia

**Keywords:** TIPS, Interventional, Portal hypertension, Parallel shunt

## Abstract

**Background:**

Transjugular intrahepatic portosystemic shunt (TIPS) is an established intervention to treat complicated portal hypertension refractory to medical or endoscopic management. TIPS dysfunction results in the recurrence of portal hypertension symptoms. In cases of TIPS dysfunction or persistent portal hypertension despite a patent primary TIPS, the creation of parallel TIPS may be the only intervention to effectively reduce portal pressure. Since the introduction of dedicated TIPS stents (Viatorr®) the incidence of TIPS dysfunction has reduced profoundly. Nevertheless, the creation of a parallel TIPS can still be necessary in the current dedicated TIPS stent era.

**Case presentation:**

We report one such patient who experienced ongoing portal hypertension induced upper gastro-intestinal haemorrhage despite multiple TIPS revisions and a patent primary TIPS.

**Conclusion:**

Following creation of a parallel TIPS, the patient remains in clinical remission with no further bleeding.

## Background

Transjugular intrahepatic portosystemic shunt (TIPS) is recommended and highly effective in appropriately selected patients with complications of portal hypertension such as refractory ascites, hydrothorax or severe variceal bleeding. TIPS dysfunction commonly results from shunt stenosis, occlusion or insufficient reduction in portosystemic gradient (PSG). This may be managed through balloon angioplasty, relining with stents or by extending the TIPS. (Alwarraky et al. 2020) In the event of ongoing portal hypertension complications with a patent primary TIPS or persistent TIPS dysfunction that is not amenable to recanalization, creation of a parallel TIPS is considered a technically challenging but viable alternative to achieve portal decompression. (Alwarraky et al. 2020) We report a patient who was successfully treated with parallel TIPS insertion for ongoing portal hypertension induced upper gastro-intestinal haemorrhage despite a patent primary TIPS and PSG under 10 mmHg.

## Case presentation

A 55-year-old female with a history of bipolar affective disorder and type 2 diabetes mellitus presented to the emergency department with haematemesis and melaena secondary to variceal haemorrhage. The patient was hypotensive and tachycardic with a serum haemoglobin of 71 g/L. Abdominal ultrasound showed a cirrhotic liver, with a subsequent diagnosis of non-alcoholic steatohepatitis cirrhosis complicated by variceal haemorrhage. At initial presentation the patient was categorised as Child Pugh A with a model for end-stage liver disease (MELD) score of 10.

**Fig. 1:**
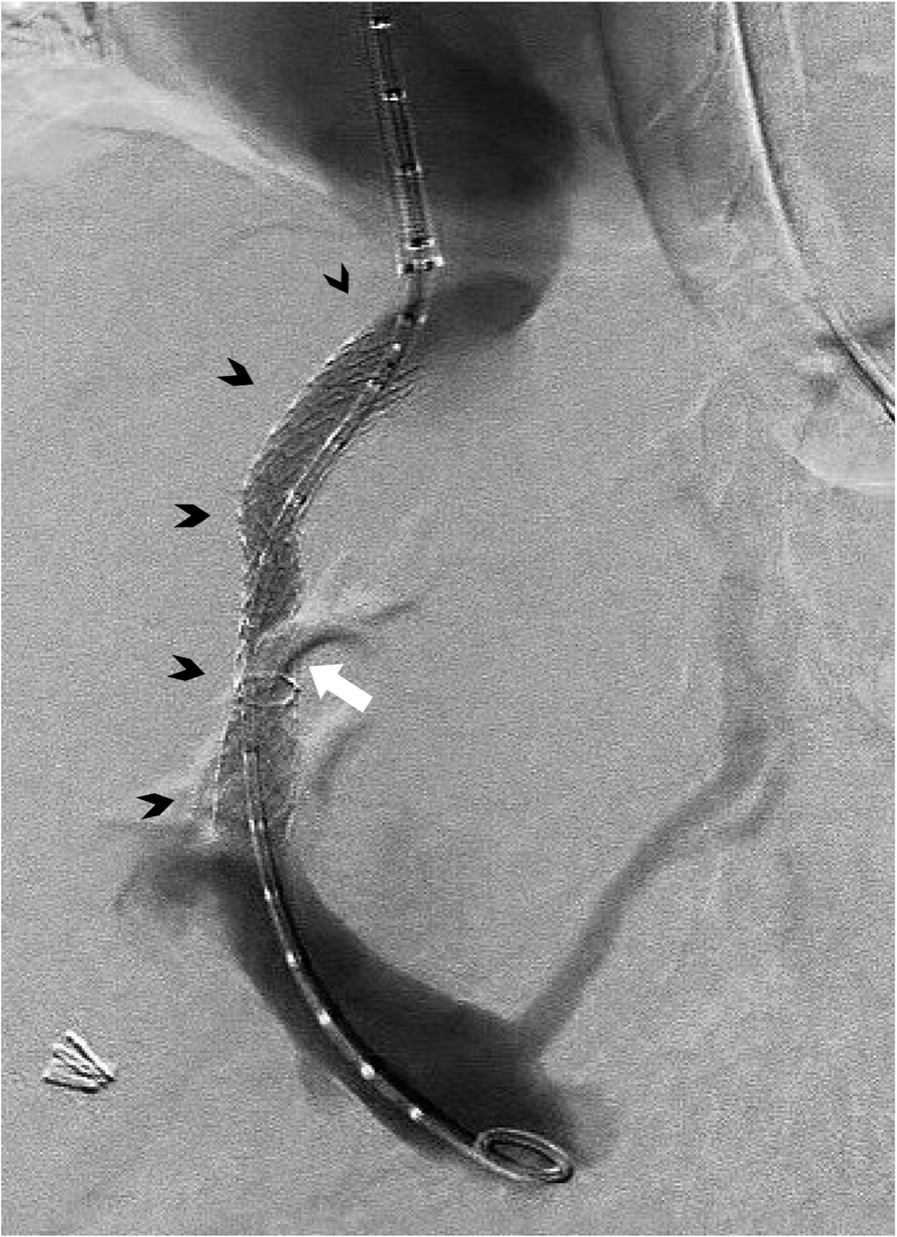
Primary TIPS extending from right hepatic vein to left portal vein with Viatorr stent in situ *(arrowheads).* Venography confirms a widely patent shunt. Extravascular contrast is obscuring part of the stent at the portal entrance *(arrow)*

Gastroscopy demonstrated grade I oesophageal varices and a large clot in the gastric fundus. A Sengstaken-Blakemore tube was inserted but failed to achieve haemostasis. Following a multidisciplinary team discussion, the patient was referred for TIPS creation. The shunt was created from the proximal right hepatic vein to the left portal vein with a Viatorr stent (Gore, Flagstaff AR, USA) extending to the hepatocaval junction, dilated to 9 mm (Fig. 1). A large coronary vein was also embolised.

**Fig. 2:**
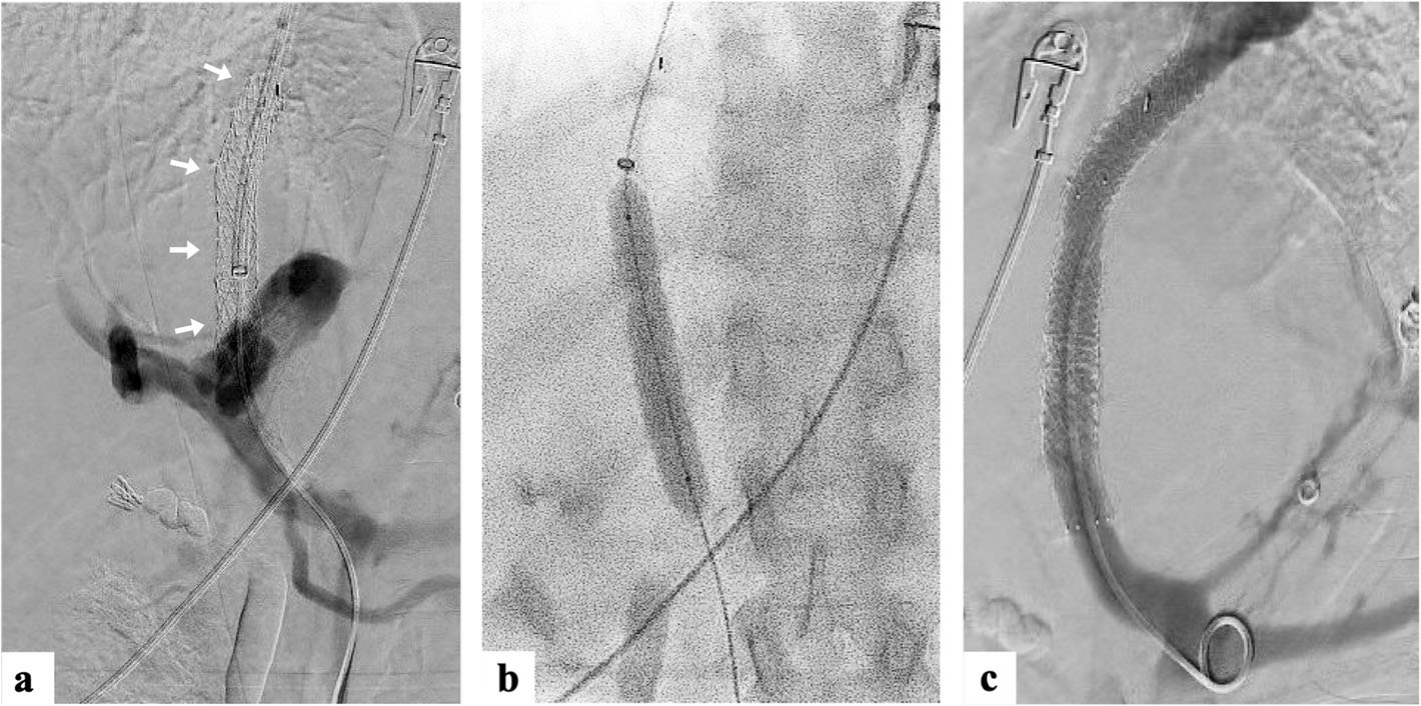
Venography of the first revision of the primary TIPS demonstrating (**a**) absence of flow through an occluded stent *(arrows)* due to thrombosis; (**b**) angioplasty with a fully inflated balloon; (**c**) successful recanalization of the TIPS with restored flow post angioplasty and further stent insertion

The patient re-presented two months later with acute variceal bleeding due to TIPS thrombosis. After balloon angioplasty failed to restore flow, the TIPS was re-lined and extended further into the portal vein (Fig. 2). Residual coronary veins were embolised via the TIPS and the patient was commenced on prophylactic direct oral anticoagulation (Apixaban).

**Fig. 3:**
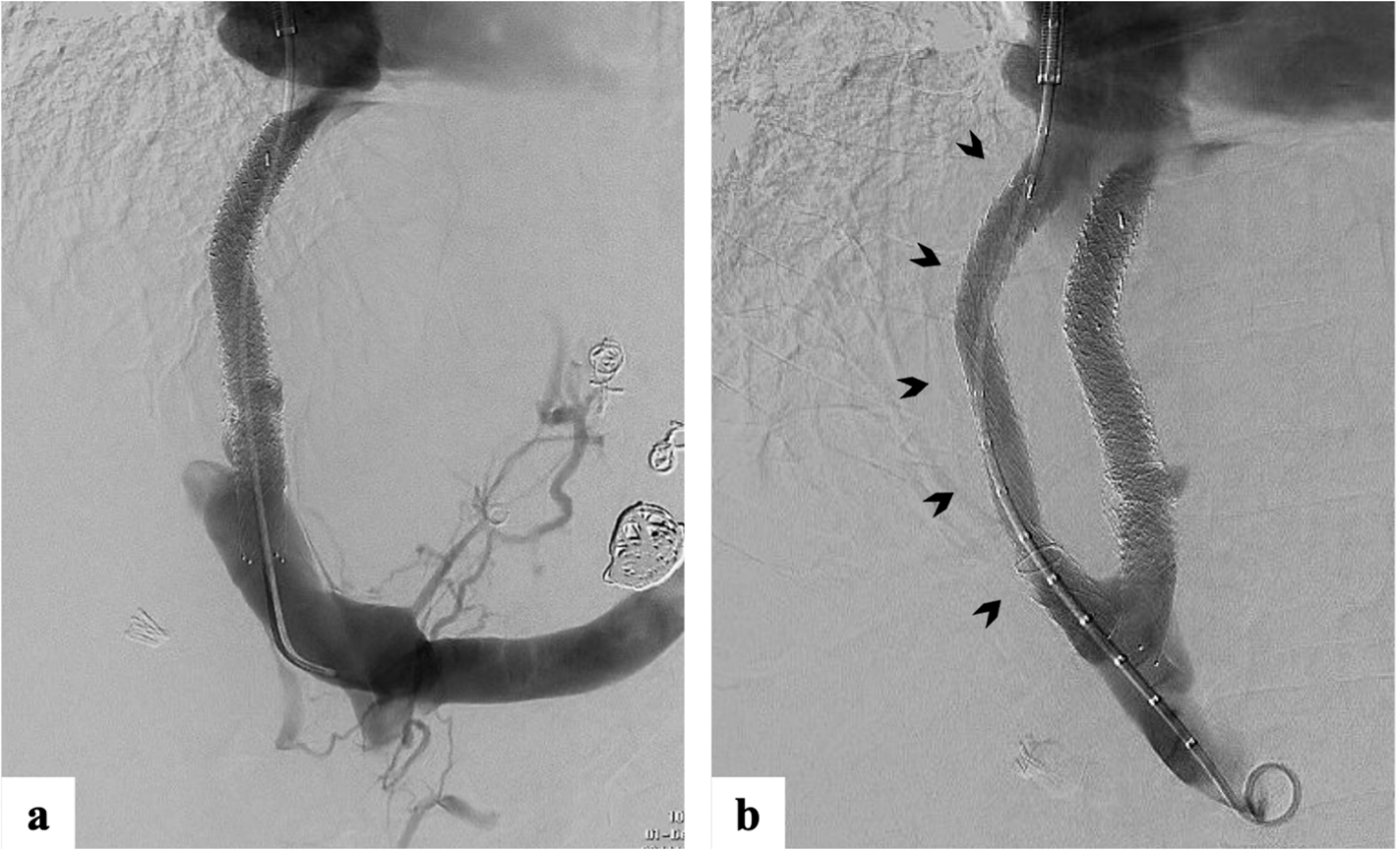
Venogram during the parallel TIPS procedure demonstrating (**a**) a patent primary shunt; (**b**) the newly created parallel TIPS *(arrowheads)* extending from the right hepatic vein to the right portal vein alongside the primary TIPS

In the months following, the patient experienced ongoing bleeding from severe portal hypertensive gastropathy despite a total of three TIPS revisions and two gastric variceal embolisations. During this period the PSG never exceeded 10 mmHg. Following MDT review, she was referred for parallel TIPS. The existing TIPS was noted to be patent with a PSG of 9 mmHg prior to creation of the parallel TIPS from the right hepatic vein to the right main portal vein. A Viatorr TIPS stent (8 cm PTFE-covered, 2 cm bare metal) (Gore, Flagstaff, USA) was inserted and dilated to 8 mm, reducing the PSG to 4 mmHg (Fig. 3).

The patient had an uneventful recovery with no immediate or delayed hepatic encephalopathy. Ultrasonography at both seven days and 5 months as well as MRI ten months post-procedure demonstrated patent parallel and primary TIPS. At ten months post-intervention, she remains well with no further bleeding or hepatic encephalopathy.

This study, being a case report, did not require institutional review board approval at the participating institution. Written informed consent was obtained from the patient for publication of this case report and any accompanying images.

## Discussion

TIPS is considered mainstay in managing complications from portal hypertension. However, ever since the first TIPS in 1969, the long-term efficacy of the intervention has been impeded by shunt dysfunction. (Rösch et al. 1969) The introduction of PTFE-covered stents replacing the use of bare metal stents has significantly improved longevity, yet patients often require multiple revisions of their TIPS (Triantafyllou et al. 2018). The major causes of bare metal stent TIPS dysfunction are in-stent thrombosis, intimal hyperplasia of the outflow hepatic vein and pseudointimal hyperplasia within the shunt lumen caused by biliary leakage from disrupted bile ducts. (Fanelli 2014) This may manifest as variceal bleeding, ascites, hydrothorax or hepatic encephalopathy, secondary to portal hypertension.

Parallel TIPS was first reported by Dabos et al. in 1998, prior to the availability of dedicated covered TIPS stents. The study assessed balloon angioplasty, re-stenting and creation of a parallel TIPS as interventions for TIPS dysfunction, recommending parallel TIPS for patients with early shunt dysfunction or severe recurrent pseudointimal hyperplasia. (Dabos et al. 1998) Parallel TIPS has since been predominantly reserved for when the primary TIPS is inaccessible or unfavourable for recanalization and for persisting portal hypertension despite verified patency on imaging. (Raissi et al. 2019) It has demonstrated similar patency rates in the mid-term compared to TIPS of patients who do not undergo parallel TIPS. (Raissi et al. 2019; Helmy et al. 2006)

Aside from the introduction of dedicated, covered TIPS stents, several factors contribute to maximising TIPS function. Some reports have suggested that entry of the stent at the left portal vein branch, compared to the right, is associated with improved longevity due to the straighter trajectory to the hepatic vein that allows for less turbulent blood flow. Puncturing the left portal vein may be more technically difficult given its location relative to the inferior vena cava. (Alwarraky et al. 2020; Chen et al. 2009; Luo et al. 2019; Chu et al. 2002) A study by Clark et al. also demonstrated longer patency in stents that extend to the hepatocaval junction rather than those terminating at the hepatic vein. (Clark et al. 2004) The TIPS stent in our patient terminated at the hepatic vein which could have contributed to the early stent occlusion in just over two months, requiring re-lining of the stent and commencement of apixaban. The use of prophylactic anticoagulation and antiplatelet therapy can reduce the incidence of both TIPS stenosis as well as de novo portal vein thrombosis occurring after TIPS. However, this carries the increased risk of rebleeding from varices. As such, certain patients may benefit from variceal embolization alongside parallel TIPS creation. (Tang et al. 2017)

He et al. describe a cohort of 10 patients undergoing parallel TIPS with non-TIPS-dedicated covered stents. (He et al. 2014) Nine patients suffered from persistent ascites and one patient had ongoing variceal bleeding despite patent primary TIPS. Reduction in PSG occurred in all patients, as well as clinical remission from portal hypertensive symptoms.

There is limited literature on parallel TIPS in which Viatorr stents, dedicated covered TIPS stents, are used for both procedures. One case report describes a patient with alcoholic cirrhosis who had two TIPS revisions before a parallel TIPS was inserted from the right hepatic to right portal vein. The PSG was reduced from 10 mmHg to 5 mmHg, however no follow-up data was included to describe the longevity of the parallel TIPS. (Larson et al. 2016) Another study reported two patients who experienced recurrent ascites, hydrothorax and elevated PSG despite venography indicating a patent primary TIPS. Both individuals demonstrated clinical improvement after parallel TIPS creation. (Parvinian and Gaba 2013) More recently, a case series by Raissi et al. followed three patients with failed endoscopic management of variceal bleeding after initial TIPS placement. Two of these patients also had patent TIPS and a PSG below 10 mmHg prior to parallel shunt insertion, comparable to our patient. (Raissi et al. 2019) Our findings concur with these latter two reports, suggesting that shunt patency may not necessarily correlate with the clinical features of TIPS dysfunction that necessitate parallel TIPS creation.

Hepatic encephalopathy is the most common complication of parallel TIPS. In most cases, this is adequately controlled with medical management. Rarer complications include biliary puncture, intraperitoneal haemorrhage and hepatic failure. (Alwarraky et al. 2020; Raissi et al. 2019) Overall, however, the parallel TIPS is considered a safe intervention for managing refractory portal hypertension, with a similar safety and adverse effect profile to a primary TIPS.

## Conclusion

Parallel TIPS still has a place in the management of persistent complications of portal hypertension in patients with patent primary TIPS created with dedicated, covered Viatorr® TIPS stents and a porto-systemic gradient below 10 mmHg.

## Data Availability

Not applicable.

## Abbreviations

TIPS: Transjugular intrahepatic porto-systemic shunt
PSG: Porto-systemic gradient
MELD: Model for end stage liver disease
PTFE: Polytetrafluoroethylene
